# Mitochondrial DNA copy number in peripheral blood of IgA nephropathy: a cross-sectional study

**DOI:** 10.1080/0886022X.2023.2182133

**Published:** 2023-03-07

**Authors:** Jiaqi Liu, Rong Wang, Ning Luo, Zhibin Li, Haiping Mao, Yi Zhou

**Affiliations:** a Department of Nephrology, The First Affiliated Hospital, Sun Yat-sen University, Guangzhou, China; b NHC Key Laboratory of Clinical Nephrology (Sun Yat-Sen University) and Guangdong Provincial Key Laboratory of Nephrology, Guangzhou, China; c Epidemiology Research Unit, The First Affiliated Hospital of Xiamen University, Xiamen, China

**Keywords:** IgA nephropathy, mitochondrial DNA, renal function, renal pathology, Oxford classification

## Abstract

Mitochondrial DNA (mtDNA) copy number (CN) is a biomarker of mitochondrial function and has been reported associated with kidney disease. However, its association with IgA nephropathy (IgAN), the most common cause of glomerulonephritis (GN), has not been evaluated. We included 664 patients with biopsy-proven IgAN and measured mtDNA-CN in peripheral blood by multiplexed real-time quantitative polymerase chain reaction (RT-qPCR). We examined the associations between mtDNA-CN and clinical variables and found that patients with higher mtDNA-CN had higher estimated glomerular filtration rate (eGFR) (*r* = 0.1009, *p* = .0092) and lower serum creatinine (SCr), blood urea nitrogen (BUN), and uric acid (UA) (*r*=−0.1101, −0.1023, −0.07806, respectively, all *p* values <.05). In terms of pathological injury, mtDNA-CN was higher in patients with less mesangial hypercellularity (*p* = .0385, *M*0 vs. *M*1 score by Oxford classification). Multivariable logistic regression analyses also showed that mtDNA-CN was lower for patients with moderate to severe renal impairment (defined as eGFR < 60 mL/min/1.73 m^2^) vs. mild renal impairment, with the odds ratio of 0.757 (95% confidence interval: 0.579–0.990, *p* = .042). In conclusion, mtDNA-CN was correlated with better renal function and less pathological injury in patients with IgAN, proposing that systemic mitochondrial dysfunction may be involved in or reflect the development of IgAN.

## Introduction

Mitochondria are the most important organelles in mammals, in which adenosine triphosphate (ATP) is generated through oxidative phosphorylation (OXPHOS) for tissue metabolism. Mitochondrial DNA (mtDNA) encodes 13 essential components involved in respiration and OXPHOS [1]. Mitochondrial dysfunction impairs cell responses to varieties of metabolic processes and dynamics of mitochondria, contributing to the pathogenesis of many common diseases, such as diabetes, obesity, cardiovascular diseases, and acute kidney disease [2–6].

Mitochondrial DNA copy number (mtDNA-CN) is a biomarker of mitochondrial function that facilitates dynamic detection and monitoring [7]. Recently, mtDNA-CN has been significantly associated with clinical feature in a broad range of clinical disorders involving the kidney damage, such as diabetic nephropathy (DN), chronic kidney disease (CKD), and incident of microalbuminuria [3,4,8–10]. Lower mtDNA-CN was reported in 83 patients with DN compared to 45 diabetes patients without kidney disease (DC) by a case-control study in Bahrain [8]. In the study of the Atherosclerosis Risk in Communities (ARIC), higher mtDNA-CN in peripheral blood was correlated with lower incident of eGFR decline [9]. It was also shown that higher mtDNA-CN was associated with lower prevalence of microalbuminuria in a cross-sectional community-based study of 694 individuals in Korea [10].

Previous studies have focused on kidney injury, while there are lack of research on the association of mtDNA-CN and specific etiology of primary glomerulonephritis (GN). IgA nephropathy (IgAN) was recognized as the most common primary GN worldwide, of whom the association with mtDNA-CN in peripheral blood has not been discovered. IgAN is diagnosed in 1–10 out of every 100,000 people each year [11,12]. The mortality of patients with IgAN is increased by 53% and the life expectancy of them is reduced by more than 6 years compared with healthy people [13]. It is reported that 40% of IgAN patients had progressed to end stage renal disease (ESRD) within 20 years, being a leading cause of ESRD in the word [5]. The deterioration of renal function is found to be a severe risk factor for progression to ESRD in IgAN [14,15]. Additionally, the risk of progression to ESRD was much higher for eGFR declined below 60 mL/min/1.73 m^2^ in IgAN [16]. At present, prediction of prognosis and diagnosis of IgAN are limited to kidney biopsy, which cannot be performed periodically due to its clinical contraindications and risk of bleeding and other clinical complications. An effective and convenient measure to evaluation of disease status such as renal function and pathological changes is required to explore in IgAN, which can be operated regularly. Given the above association of mtDNA-CN with kidney damage and adverse renal outcomes [3,4,9,10], we aimed to investigate the specific association of mtDNA-CN in peripheral blood with IgAN manifestations. In our present study, we depicted for the first time the association of mtDNA-CN with the clinical and pathological features in a large number of biopsy-diagnosed IgAN patients.

## Methods

### Study population

**Figure 1.**
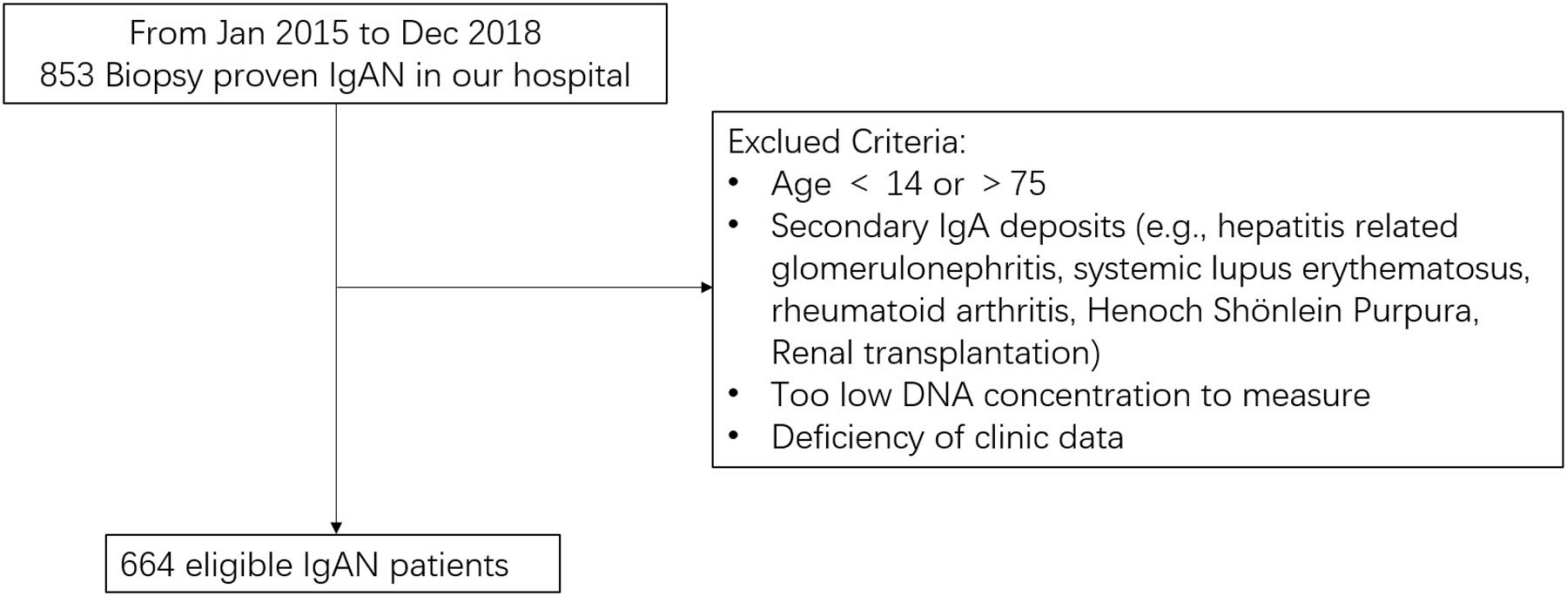
The protocol for selection of IgAN patients.

In the current study, 853 individuals diagnosed as IgAN by kidney biopsy were enrolled from the First Affiliated Hospital of Sun Yat-Sen University from January 2015 to December 2018. Blood samples and clinical phenotype data were collected subsequently. Patients were excluded if one or more of the following criteria was met: age <14 or >75; secondary IgA deposits (e.g., hepatitis related GN, systemic lupus erythematosus, rheumatoid arthritis, Henoch-Schönlein purpura, and renal transplantation); too low DNA concentration to measure; deficiency of clinic data. Finally, 664 eligible patients were enrolled in this study (Figure 1). Patients were classified into two groups: mild renal impairment (eGFR ≥ 60 mL/min/1.73 m^2^) or moderate to severe renal impairment (eGFR < 60 mL/min/1.73 m^2^). According to the Oxford classification, the pathological severity in IgAN patients was checked [17]. This study was approved by the Human Research Ethics Committee of the First Affiliated Hospital of Sun Yat-Sen University, Guangzhou, China (no. 201037). All participants provided written informed consent.

### Clinical and laboratory data

Clinical data were obtained from the department of clinical laboratory, the First Affiliated Hospital of Sun Yat-Sen University. Peripheral bloods were obtained in 664 participants at the time of kidney biopsy. Serum creatinine (SCr), blood urea nitrogen (BUN), and uric acid (UA) were measured by standard procedures. To determine proteinuria levels, 24 h urine was collected. Estimated glomerular filtration rate (eGFR) was computed by the Chronic Kidney Disease Epidemiology Collaboration (CKD-EPI) method [18]. Kidney biopsies were scored by professional pathologists referring to Oxford MEST-C Classification [19]. Specifically, *M* was scored in terms of mesangial hypercellularity: when >50% of glomeruli showing four or more cells in one or more mesangial area, not including central core and region of the vascular pole were scored *M*1. *E* score or *S* score was defined as absent (0) or present (1) of endocapillary hypercellularity (E) or segmental glomerulosclerosis (S). *T* score was defined in terms of estimated percentage of interstitial fibrosis and tubular atrophy: *T*0 (≤25%), *T*1 (26–50%), and *T*2 (>50%). *C* score was referred to crescents: *C*0 (0%), *C*1 (0% to <25%), and *C*2 (>25%).

### Measurement of mtDNA-CN in peripheral blood

Measurement of mtDNA-CN has been described previously [20]. mtDNA genes ND1 in peripheral blood were assessed by real-time quantitative polymerase chain reaction (RT-qPCR). DNA was isolated from blood samples using QIA symphony DSP DNA Midi Kit (Qiagen, Valencia, CA) and concentrations were detected by using ABI TaqMan chemistry (Applied Biosystems, Waltham, MA). Specimens were performed in triplicate for each assay. The mitochondrial target (ND1) (assay ID Hs02596873_s1) and nuclear target (RPPH1) (assay ID Hs03297761_s1) of cycle thresholds (Cts) were detected. Each sample was run in triplicate on a 384-well plate in a 10-μL reaction containing 20 ng of DNA. The thermal profile was set up according to manufacturer’s instructions (Applied Biosystems, Waltham, MA, Cat#4444557) as follows: 50 °C for 2 min, 95 °C for 2 min, and 40 cycles of 95 °C for 3 s, and 60 °C for 30 s. Cycle threshold value was determined from the amplification curve for each target by the ABI Viia7 software. The difference in Cts between the two genes (ΔCt) was computed for each well as the difference between the Ct for the RPPH1 target and the Ct for the ND1 target, as a measure of mtDNA-CN relative to nuclear DNA copy number. Quality control procedures were performed as follows: excluding outliers from the triplicate assays when the SD of ΔCt was >0.5, and kicking out a replicate with Ct for ND1 of >30, a Ct for RPPH1 of >5 SDs, and ΔCt of >3 SDs. We defined SD (with a mean of 0) as the unit of standardized determination for mtDNA-CN as previous [20]. ND1 stands for nicotinamide adenine dinucleotide dehydrogenase subunit-1. mtDNA-CN was dichotomized using a study-specific median measurement level as the cutoff to define ‘high mtDNA-CN’ as an measurement level at or above the median vs. ‘low mtDNA-CN’ as an measurement level below the median.

### Statistical analysis

Baseline characteristic data were expressed as mean ± standard deviation for continuous variables or *n* (%) for categorical variables. Median (IQR) was displayed for non-normal variables. Differences of baseline characteristics between subjects (categorized by mtDNA-CN, eGFR, or *M* scores of the Oxford classification) were analyzed by Student’s *t*-test for normal variables, Wilcoxon’s rank sum test for non-normal variables, and Chi-square test for categorical variables. Measurement of mtDNA-CN was expressed as standardized residuals. Normality tests were conducted by Shapiro–Wilk’s test and found data following approximation of normal distribution. Pearson’s correlation analysis was used to explore correlations of mtDNA-CN in peripheral blood with eGFR, SCr, BUN, UA, and proteinuria. Multivariable logistic regression model was used to evaluate associations of mtDNA-CN with mild vs. moderate to severe renal impairment with adjustment for the potential confounding variables. Adjusted odds ratio (OR) and 95% confidence intervals (CIs) were applied to quantify the association of the mtDNA-CN with renal impairment in IgAN. All *p* values were two-sided and *p*< .05 was taken as statistically significant. Statistical analyses were performed in SPSS 25 software, version 14 (SPSS Inc., Chicago, IL).

## Results

### Characteristics of patients with IgAN Stratified by mtDNA-CN

Table 1 shows the characteristics of the enrolled 664 patients with IgAN stratified by mtDNA-CN with overall mean of 0.0077. mtDNA-CN was dichotomized using a study-specific median level as the cutoff to define ‘high mtDNA-CN’ with mean of −0.483 and ‘low mtDNA-CN’ with mean of 0.467. In the higher mtDNA-CN group, clinical indicators of SCr and BUN were lower (*p* = .0129 and .0057, respectively), while eGFR levels were higher (*p* = .0167). In the high mtDNA-CN group, the mean of SCr was 161.9 μmol/L and BUN was 7.591 mmol/L and eGFR was 66.52 mL/min/1.73 m^2^. In the low mtDNA-CN group, the mean of SCr was 195.4 μmol/L and BUN was 8.697 mmol/L and eGFR was 60.01 mL/min/1.73 m^2^. The proteinuria, UA, and histological scores from IgAN patients had no significant difference at different levels of mtDNA-CN.

**Table 1.** Characteristics of patients with IgAN stratified by mtDNA-CN.

Characteristics	mtDNA-CN (low)	mtDNA-CN (high)	*p* Value
mtDNA-CN (SD)	–0.4828 ± 0.4646	0.4673 ± 0.3000	.0001*
Sex (male), *n* (%)	176 (52.85%)	168 (50.76%)	.6418
Age, years	36.14 ± 10.64	36.24 ± 10.34	.8971
Estimated GFR (mL/min/1.73 m^2^)	60.01 ± 35.43	66.52 ± 34.40	.0167*
Serum creatinine (μmol/L)	195.4 ± 190.6	161.9 ± 153.9	.0129*
Proteinuria (g/24 h)	2.262 ± 2.408	2.002 ± 2.177	.1439
Blood urea nitrogen (mmol/L)	8.697 ± 5.655	7.591 ± 4.553	.0057*
Uric acid (μmol/L)	444.3 ± 120.6	430.6 ± 108.8	.1232
*Oxford classification* ^a^			
Mesangial hypercellularity (*M*1), *n* (%)	218 (71.01%)	197 (63.96%)	.0622
Endocapillary hypercellularity (*E*1), *n* (%)	59 (19.22%)	69 (22.40%)	.3316
Segmental glomerulosclerosis (*S*1), *n* (%)	184 (59.93%)	187 (60.71%)	.7800
Tubular atrophy/interstitial fibrosis (*T*1,*T*2), *n* (%)	135 (43.97%)	122 (39.61%)	.4414
Crescents (*C*1,*C*2), *n* (%)	188 (61.24%)	187 (60.71%)	.5070

eGFR: estimated glomerular filtration rate; SCr: serum creatinine; BUN: blood urea nitrogen; UA: uric acid; mtDNA: mitochondrial DNA; CN: copy number.

Data are shown as mean ± standard deviation for continuous variables or *n* (%) for categorical variables. SD (with a mean of 0) as the unit of standardized determination for mtDNA-CN.

^a^ Forty-nine patients without complete Oxford classification are excluded.

* *p*-value < 0.05.

### Correlations of mtDNA-CN in peripheral blood with renal function

**Figure 2.**
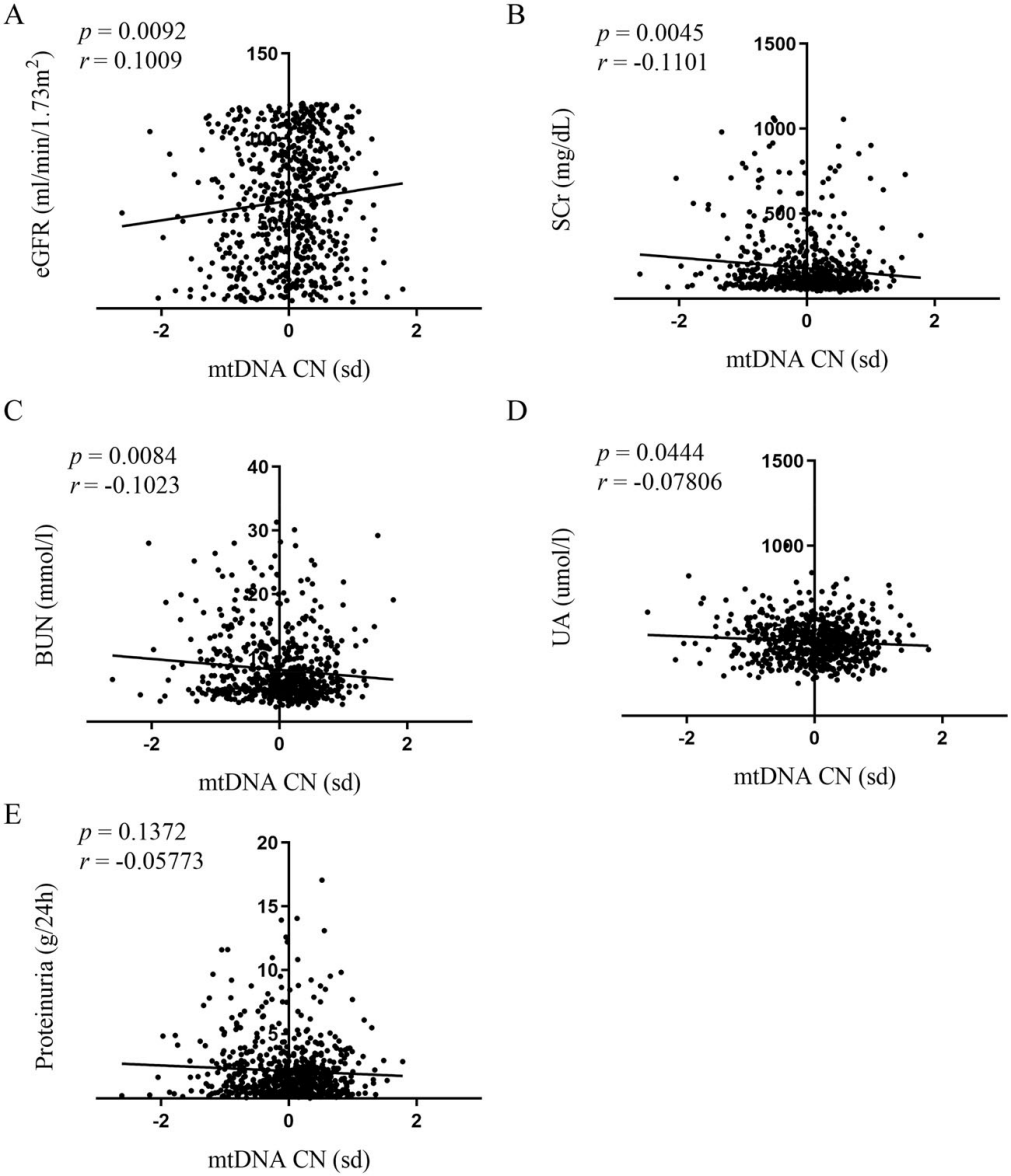
Correlations of mtDNA-CN in peripheral blood with renal function. Association between mtDNA-CN in peripheral blood and eGFR (A), SCr (B), BUN (C), UA (D), and proteinuria (E) were analyzed by Spearman’s rank correlation analysis. *N* = 664. eGFR: estimated glomerular filtration rate; mtDNA: mitochondrial DNA; CN: copy number. SD (with a mean of 0) as the unit of standardized determination for mtDNA-CN.

In correlation measurements of mtDNA-CN with clinical indicators of renal function, we analyzed the eGFR, SCr, BUN, and UA. The data showed that higher mtDNA-CN was associated with higher eGFR (*p* = .0092, Figure 2(A)) and lower SCr, BUN, and UA (*p* = .0045, Figure 2(B); *p* = .0084 Figure 2(C) and *p* = .0444, Figure 2(D)). The correlations were statistically significant, but their coefficients were small (*r* = 0.1009, Figure 2(A); *r*=−0.1101, Figure 2(B); *r*=−0.1023, Figure 2(C); and *r*=−0.07806, Figure 2(D)).

### mtDNA-CN characteristics of patients with IgAN stratified by eGFR

**Figure 3.**
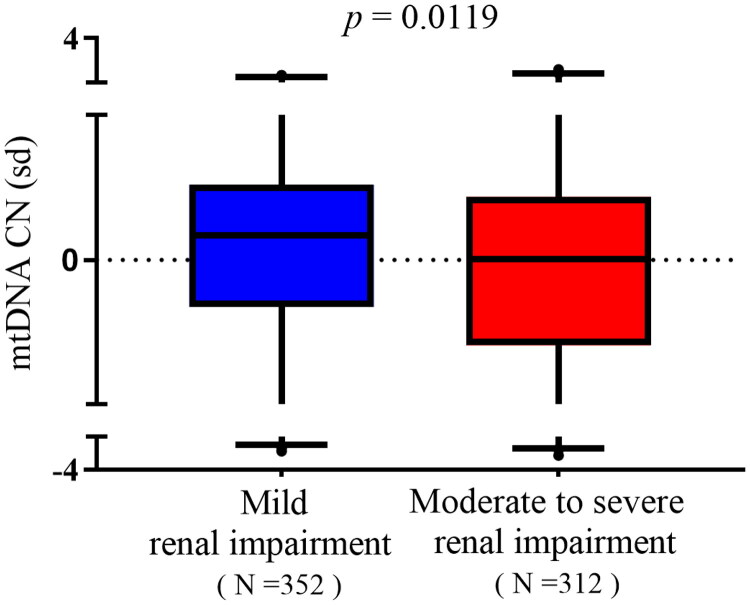
mtDNA-CN in patients with IgAN stratified by eGFR. Data were analyzed by Student’s *t*-test. mtDNA: mitochondrial DNA; CN: copy number. SD (with a mean of 0) as the unit of standardized determination for mtDNA-CN.

Enrolled patients with IgAN were divided into two groups according to the higher risk of severe clinical outcome in patients with eGFR declined below 60 mL/min/1.73 m^2^ in IgAN [16,21]: (1) mild renal impairment (eGFR ≥ 60 mL/min/1.73 m^2^, *n* = 352) and (2) moderate to severe renal impairment (eGFR < 60 mL/min/1.73 m^2^, *n* = 312). mtDNA-CN was significantly higher in patients with mild renal impairment compared with those of moderate to severe renal impairment (0.04872 vs. −0.07151, *p* = .0119, Figure 3). In mild renal impairment group, renal function of patients was better (all *p*< .001, Table 2) and pathological injury such as endocapillary hypercellularity and interstitial fibrosis/tubular atrophy were less (*p* = .009 and *p*< .001, respectively, Table 2).

**Table 2.** mtDNA-CN characteristics of patients with IgAN stratified by eGFR.

Characteristics	EGFR ≥60 (mL/min/1.73 m^2^) (*n* = 352)	EGFR <60 (mL/min/1.73 m^2^) (*n* = 312)	*p* Value
Sex (male), *n* (%)	84 (50.00%)	221 (53.25%)	.1153
Age, years	34.19 ± 9.361	38.45 ± 11.22	.0001*
Serum creatinine (μmol/L)	84.80 ± 19.88	284.6 ± 206.9	.0001*
Proteinuria (g/24 h)	1.477 ± 1.818	2.871 ± 2.547	.0001*
Blood urea nitrogen (mmol/L)	5.107 ± 1.375	11.57 ± 5.691	.0001*
Uric acid (μmol/L)	390.2 ± 89.42	490.8 ± 117.3	.0001*
*Oxford classification* ^a^			
Mesangial hypercellularity (*M*1), *n* (%)	109 (32.83%)	91 (32.15%)	.8588
Endocapillary hypercellularity (*E*1), *n* (%)	276 (83.13%)	211 (74.55%)	.0091*
Segmental glomerulosclerosis (*S*1), *n* (%)	142 (42.77%)	104 (36.74%)	.1078
Tubular atrophy/interstitial fibrosis (*T*1,*T*2), *n* (%)	300 (90.36%)	237 (83.74%)	.0006*
Crescents (*C*1,*C*2), *n* (%)	311 (93.67%)	266 (93.99%)	.2258

eGFR: estimated glomerular filtration rate; SCr: serum creatinine; BUN: blood urea nitrogen; UA: uric acid; mtDNA: mitochondrial DNA; CN: copy number.

Data are shown as mean ± standard deviation for continuous variables or *n* (%) for categorical variables. SD (with a mean of 0) as the unit of standardized determination for mtDNA-CN.

^a^ Forty-nine patients without complete Oxford classification are excluded.

* *p*-value < 0.05.

### Multivariable logistic regression analyses of associations between mtDNA-CN and renal impairment

Based on the significant difference of mtDNA-CN between two groups with different degrees of renal impairment, multivariable logistic regression analyses of mtDNA-CN on renal impairment were performed. Table 3 shows that the significant variables of aggravating renal impairment (eGFR < 60 mL/min/1.73 m^2^) were lower mtDNA-CN (adjusted OR: 0.757, 95% CI: 0.579–0.990, *p* = .042) and higher proteinuria (OR = 1.436, 95% CI: 1.296–1.591, *p*< .001), adjusting for known potential confounding variables (sex, age, and proteinuria).

**Table 3.** Multivariable logistic regression analyses of associations between mtDNA-CN and renal impairment.

Variables	*p*	OR	95% CI
mtDNA-CN (SD)	.042	0.757	(0.580, 0.983)
Proteinuria (g/24 h)	<.001	1.436	(1.287, 1.571)
Sex	.042	0.709	(0.510, 0.987)
Age	<.001	1.045	(1.027, 1.062)

Variables were identified by using exploratory univariable analyses with explanatory variables entered into the multivariable stepwise logistic regression model. *N* = 664. SD (with a mean of 0) as the unit of standardized determination for mtDNA-CN.

### Relationships between mtDNA-CN and renal pathological changes in IgAN patients

**Figure 4.**
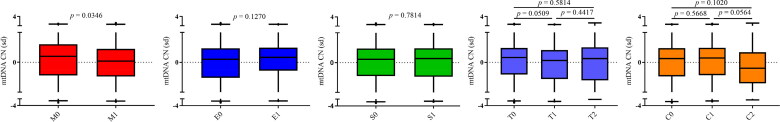
Relationships between mtDNA-CN and renal pathological changes in IgAN patients. Data were analyzed by Student’s *t*-test. SD (with a mean of 0) as the unit of standardized determination for mtDNA-CN. *N* = 615. Forty-nine individuals were excluded due to incomplete pathological Oxford classification information. Renal pathological changes were scored by professional pathologists referring to Oxford MEST-C Classification. Patients were scored *M*0 or *M*1 in terms of mesangial hypercellularity: when >50% of glomeruli showing four or more cells in one or more mesangial area, not including central core and region of the vascular pole were scored *M*1. *E* score or *S* score were defined as absent (0) or present (1) of endocapillary hypercellularity (E) or segmental glomerulosclerosis (S). *T* score was defined in terms of estimated percentage of interstitial fibrosis and tubular atrophy: *T*0 (≤25%), *T*1 (26–50%), and *T*2 (>50%). *C* score referred to crescents: *C*0 (0%), *C*1 (0% to <25%), and *C*2 (>25%).

The Oxford classification has been accepted by the majority of clinicians and investigators as an international consensus for pathological classification of IgAN. It includes the MEST-C score, referring to mesangial hypercellularity (M), endocapillary hypercellularity (E), segmental glomerulosclerosis (S), tubular atrophy/interstitial fibrosis (T), and crescents (C). Among them, the *M*0 score refers to the mesangial hypercellularity in glomeruli < 50%, while the *M*1 score refers to ≥50% of mesangial hypercellularity. Our data showed that mtDNA-CN was markedly higher in patients with less mesangial hypercellularity (*M*0 vs. *M*1 score by Oxford classification, *p* = .0346, Figure 4). mtDNA-CN appeared to be trending higher in patients with *T*0 compared to *T*1 score, and also higher in patients with *C*1 compared to *C*2 score, even though no statistical significance was found (*p=* .0509 and .0564, respectively, Figure 4). However, comparisons in terms of *E* score and *S* score found no difference. These results indicated that mtDNA-CN could reflect the mesangial hypercellularity in glomeruli which is the early pathological injury in the progression of kidney disease. Supplemental Table 1 shows the baseline characteristics of patients stratified by *M* score. Notably, there was no significant difference in age, eGFR, SCr, proteinuria, BUN, and UA between patients with *M*0 and *M*1 score (all *p*> .05).

## Discussion

In the present study of 664 patients with IgAN, we found that mtDNA-CN in peripheral blood was associated with renal function and inversely associated with pathological injury. The higher mtDNA-CN is associated with the better renal function having higher eGFR, lower SCr, BUN, and UA and the less pathological change of mesangial hypercellularity.

Recently, an increasing number of studies have reported the significant association of mtDNA-CN with various clinical disorders, worthy of more attention in a broader clinical practice. In a study of Black and White individuals from Chronic Renal Insufficiency Cohort (CRIC), higher mtDNA-CN in peripheral blood was associated with higher eGFR [22]. It is also reported that lower mtDNA-CN was associated with higher prevalent CKD (defined as eGFR < 60 mL/min per 1.73 m^2^) in a community-based cohort of Americans [9]. When paying attention to specific etiology of primary GN, a cross-sectional study in Korea has first reported that urinary mtDNA-CN was negatively associated with eGFR in 31 patients with IgAN, but failed to identify the statistical correlations of other indicator or pathological injury [23]. Consistent with previous reports, we observed that the patients with higher mtDNA-CN in peripheral blood had the better renal function considering the higher eGFR, lower SCr, BUN, and UA. In terms of pathological change, patients with higher mtDNA-CN had the less mesangial hypercellularity in renal glomeruli. Additionally, mtDNA-CN had an inverse association of moderate to severe renal impairment with declined eGFR < 60 mL/min per 1.73 m^2^ (OR = 0.757, 95% CI = 0.579–0.990). The difference between two studies of IgAN, the Korea study of urinary mtDNA-CN and our study of mtDNA-CN in peripheral blood, was due to the different populations and different sources of mtDNA-CN may have different characteristics. It is worth pointing out that most studies have focused on the characteristic of mtDNA-CN in peripheral blood, as peripheral blood samples more credibly and systematically reflect the state of the body. Therefore, our results suggested that mtDNA-CN may be a possible marker for clinical monitoring of IgAN patients to systematically reflect the status of disease, including renal function and pathological change.

On the other hand, mtDNA-CN is a great biomarker of mitochondrial function, which can directly reflect mitochondrial function [18,24]. Mitochondrial dysfunction has been identified as underlying mechanisms for the development and progression of kidney disease, like acute kidney injury and CKD [25,26]. It is said that accumulation of mtDNA damage and the consequential decrease in mtDNA-CN was linked to kidney injury [8–10,23]. Ashar and colleagues indicated that higher mtDNA-CN is a marker of higher levels of mitochondrial replication and cellular energy reserves, with lower levels of mtDNA-CN likely reflecting mitochondrial depletion [20]. In patients with IgAN, electron microscopic images also found the mitochondrial morphological alterations in kidney, of which the mitochondria were small and disorganized [23]. These findings, in conjunction with our findings of mtDNA-CN associations with renal function and histological damage, further suggested that mitochondrial dysfunction may play an etiologic role in IgAN.

Furthermore, a few studies have examined the correlation between mtDNA-CN and adverse outcomes in the elderly individuals, patients on hemodialysis and CKD. It is reported that higher mtDNA-CN was associated with lower risk of all-cause mortality in the elderly individuals, patients on hemodialysis or with renal dysfunction [3,4,20]. Additionally, lower mtDNA-CN was associated with prevalent frailty. In some cohort studies, lower mtDNA-CN have been associated with higher risk of kidney failure (HR, 1.30; 95% CI, 1.10–1.55), which was proposed as a useful target for intervention in the progression of kidney disease [22,27]. These findings suggested that mtDNA-CN can also be a new target for intervention during the progression of IgAN.

Besides, our enrolled patients were first diagnosed with IgAN by renal-biopsy. Most of them received conventional therapy for symptom relief at collection. Of the included patients, 398 participants were not receiving immunosuppressive agent (neither steroids nor other immunosuppressants). Through comparing the levels of mtDNA-CN in patients with immunosuppressive treatment or not, we identified no significant difference by statistical analysis (*p* = .1969, data not shown). Meanwhile, we conducted the same statistical analysis as Figures 2–4 in our study for patients treated with immunosuppressive agent as well as patients treated without immunosuppressive agent. We found a similar tendency that patients with mild renal injury had higher mtDNA-CN compared with patients with more severe renal injury. These data suggested that treatment at baseline seemed not to significantly affect the levels of mtDNA-CN. In line with previous study, treatment did not affect mtDNA-CN in urine from patients with IgAN [23]. However, the question of whether treatment affects mtDNA-CN levels seems to require future longitudinal studies to be thoroughly investigated.

This study has several strengths over the previous studies. Our study was the first to report the association of mtDNA-CN in peripheral blood with clinical indicator in patients with biopsy-proven IgAN. Second, it included a large sample size of 664 IgAN patients comparing to the Korea study of 31 IgAN patients, which might provide more reliable information.

This study also has limitations. Due to the difficulty in follow-up, we only assessed correlations between mtDNA-CN and IgAN by cross-sectional study, a longitudinal analysis is required. Regarding the relative small coefficients in correlation analysis, further studies are also warranted to confirm it.

In conclusion, mtDNA-CN, as a sensitive marker of mitochondrial function, was inversely associated with clinical indicators of renal function decline and pathological injury in patients with IgAN, suggesting mitochondrion involved in pathophysiological processes of IgAN. Future studies are warranted to evaluate changes in mitochondrial function in the progression of IgAN and investigate whether intervention on mitochondrial function can improve IgAN.

## Supplementary Material

Supplemental Material
