# Supplementary material for: Mitochondrial DNA copy number in peripheral blood of IgA nephropathy: a cross-sectional study
Source: Ren Fail. 2023 Mar 7;45(1):2182133. doi: 10.1080/0886022X.2023.2182133 (PMC10013479; doi:10.1080/0886022X.2023.2182133)
Supplement: Supplemental Material [file IRNF_A_2182133_SM1914.pdf]

Supplementary Material

Supplemental Table 1. Characteristics of Patients with Different Mesangial Hypercellularity Score.

**Characteristics**	**M0 (n=200)**	**M1 (n= 415)**	***p*-value**
MtDNA-CN (sd)	0.1599(-0.3528, 0.4830)	0.03061(-0.3802, 0.3600)	0.0346*
Sex (Male),n (%)	100(50.00%)	221(53.25%)	0.4501
Age, year	35.73±10.58	39.29±10.42	0.5283
Estimated GFR (ml/min/1.73m^2)	63.90±35.43	64.21±34.53	0.9147
Serum Creatinine (um/l)	174.6 ± 158.4	173.7 ±173.7	0.8163
Proteinuria (g/24h)	1.980 ±1.960	2.193 ±2.478	0.8204
Blood Urea Nitrogen (mmol/l)	8.092 ±5.282	7.928 ± 4.878	0.9324
Urea Acid (umol/l)	440.9 ±128.8	436.7 ±107.0	0.8274
**Oxford classification[a]**			
Endocapillary hypercellularity (E1),n (%)	33(16.50%)	95(22.89%)	0.0677
Segmental glomerulosclerosis (S1),n (%)	116(58.00%)	254(61.20%)	0.4475
Tubular atrophy/Interstitial fibrosis (T1,T2),n (%)	45(22.50%)	142(34.22%)	0.2484
Crescents (C1,C2), n (%)	116(58.00%)	259(62.41%)	0.1635

Supplemental Table 1. Characteristics of Patients with Different Mesangial Hypercellularity Score.

Data were shown as mean ± standard deviation for continuous variables or n (%) for categorical variables. Median (IQR) was displayed for non-normal variables. eGFR = estimated glomerular filtration rate, SCr = serum creatinine, BUN = blood urea nitrogen, UA = urea acid, MtDNA = mitochondrial DNA, CN = copy number. sd (with a mean of 0) as the unit of standardized determination for mtDNA-CN. [a]49 patients without complete Oxford classification are excluded. *Indicate p-value < 0.05.

Information Classification: General
